# In silico evolution of the *Drosophila* gap gene regulatory sequence under elevated mutational pressure

**DOI:** 10.1186/s12862-016-0866-y

**Published:** 2017-02-07

**Authors:** Aleksandra A. Chertkova, Joshua S. Schiffman, Sergey V. Nuzhdin, Konstantin N. Kozlov, Maria G. Samsonova, Vitaly V. Gursky

**Affiliations:** 1 Systems Biology and Bioinformatics Laboratory, Peter the Great St. Petersburg Polytechnic University, Polytechnicheskaya, 29, St. Petersburg, 195251 Russia; 2 Molecular and Computational Biology, University of Southern California, Los Angeles, 90089 CA USA; 3 Theoretical Department, Ioffe Institute, Polytechnicheskaya, 26, St. Petersburg, 194021 Russia

**Keywords:** Evolution, Regulatory sequence, Gap gene regulatory network, Thermodynamic expression model, Drosophila development

## Abstract

**Background:**

*Cis*-regulatory sequences are often composed of many low-affinity transcription factor binding sites (TFBSs). Determining the evolutionary and functional importance of regulatory sequence composition is impeded without a detailed knowledge of the genotype-phenotype map.

**Results:**

We simulate the evolution of regulatory sequences involved in *Drosophila melanogaster* embryo segmentation during early development. Natural selection evaluates gene expression dynamics produced by a computational model of the developmental network. We observe a dramatic decrease in the total number of transcription factor binding sites through the course of evolution. Despite a decrease in average sequence binding energies through time, the regulatory sequences tend towards organisations containing increased high affinity transcription factor binding sites. Additionally, the binding energies of separate sequence segments demonstrate ubiquitous mutual correlations through time. Fewer than 10% of initial TFBSs are maintained throughout the entire simulation, deemed ‘core’ sites. These sites have increased functional importance as assessed under wild-type conditions and their binding energy distributions are highly conserved. Furthermore, TFBSs within close proximity of core sites exhibit increased longevity, reflecting functional regulatory interactions with core sites.

**Conclusion:**

In response to elevated mutational pressure, evolution tends to sample regulatory sequence organisations with fewer, albeit on average, stronger functional transcription factor binding sites. These organisations are also shaped by the regulatory interactions among core binding sites with sites in their local vicinity.

**Electronic supplementary material:**

The online version of this article (doi:10.1186/s12862-016-0866-y) contains supplementary material, which is available to authorized users.

## Background

Historically, the study of mathematical evolution was practiced as the study of the changes in gene frequencies, as a consequence of neutral and selective evolutionary forces. For the sake of simplicity, many of these early population genetic models analyzed these processes without considering the complications imposed by including genotype-phenotype mappings, such as descriptions of the genetic regulatory networks (GRNs) involved in development. Over the past several decades models of gene regulatory networks (of varying degrees of realism) have been developed and applied to fill this gap in evolutionary research [1–4]. Studies lacking highly detailed computational descriptions of relevant GRNs typically draw inferences based solely on sequence, and the direct properties thereof, potentially missing subtleties that can only be deduced from a systems-level approach. For instance, previous work suggests that the binding affinity of a transcription factor binding site (TFBS) only weakly predicts its phenotypic importance [5–8], contradicting the naive notion that binding site strength strongly predicts selective importance. Consistent with this view, we study the evolutionary dynamics of biological regulatory sequences employing a systems level approach. We simulate sequence evolution using an experimentally derived, computational model of a developmental network and genotype-phenotype mapping [6].

We simulate expression of four segmentation genes during early fruit fly development using a hybrid reaction-diffusion and thermodynamic computational model, fit to empirical expression patterns and to wild-type regulatory sequences. Given a sequence, TFBSs and their respective affinities are assigned, and gene product concentrations (mRNA and proteins) are calculated [6]. Both reaction-diffusion and ‘gene-circuit’ modelling approaches have been successfully applied to study gene network and enhancer evolution in *Drosophila* development [4, 9–14].

The cumulative binding energy, and thus the net impact, a regulatory sequence imposes on its target gene is a complex function of, minimally: DNA accessibility, TFBS presence and quantity, and transcription factor/TFBS affinity. As such, many different combinations of these variables, in principle, can precipitate similar, if not identical, regulatory effects. However, as the regulatory sequences are a consequence of gradual, neutral and selective evolutionary changes, subsets of these functionally-equivalent schemes may be more and less likely to emerge and more and less stable through evolutionary time, given specific historical, population, and mutational conditions. Therefore, the sets of acceptable regulatory schemes connected by either neutral or compensatory evolutionary changes are of particular interest to evolutionary biologists.

It is well documented and experimentally demonstrated that there is tremendous regulatory sequence variation and divergence among even closely related species, despite conservation of expression patterns and regulatory dynamics [15–19]. Presumably TFBS turnover and sequence divergence occurs mostly as populations traverse the set of equivalent regulatory schemes on a path connected by neutral changes. However, it is not immediately apparent what such evolutionary paths would look like, and how neutral and selective forces bias this search. Here we elaborate some of the consequences of high mutational pressure on sequence complexity.

Prior simulation studies have suggested that evolutionary paths tend to sample “complex” regulatory schemes (i.e. many, weaker TFBSs in contrast with fewer, stronger TFBSs), simply as a result of the relative frequencies of possible regulatory schemes [10]. It is further emphasized that patterns of seemingly nonrandom regulatory sequence complexity (measured in part by TFBS quantity) may simply be the result of non-adaptive processes [20, 21]. Furthermore, in order to predict the likelihood of evolutionary changes it may be helpful to catalogue both the functional importance of specific regulatory regions as well as the (in)visibility of regulatory regions (and sub-regions) to natural selection.

To add to this discussion, we study the evolutionary dynamics of a population of *Drosophila melanogaster*, characterized and assessed by the expression of its early developmental regulatory network under elevated mutational pressure, focusing our analysis on binding energy profile evolution. The developmental model employed suggests that the regulatory sequences under study are complex; composed of many weak (rather than a few strong) binding sites. As such, there is only a weak correlation between a binding site’s affinity and its impact on gene expression. We emphasize a specific question: given the small correlation between sequence binding affinity and functional importance, how can the evolutionary significance (if any) of regulatory sequence reorganisation be assessed? We believe that a quantitative answer to this question will be generalizable to systems lacking such detailed computational genotype-phenotype models.

In this simulation, we observe a quick and dramatic drop in the total quantity of TFBSs in conjunction with a positive shift in the distribution of remaining regulatory binding energies through evolutionary time. This reorganisation is partially influenced by a sequence’s binding affinity annotation specificity and redundancy. TFBSs determined to be functionally important (some with weak binding affinities) are shown to be conserved during evolution. Finally, TFBSs in close proximity to important binding sites are more likely to be maintained through evolutionary time.

## Methods

### Regulatory sequences and binding sites for gap genes

We analyzed regulatory regions of the gap genes *hunchback* (*hb*), *Krüppel* (*Kr*), *giant* (*gt*), and *knirps* (*kni*) and extracted TFBSs in these regions using the same procedure as described in [6]. The putative regulatory regions spanned 12 Kbp upstream and 6Kbp downstream of the transcription start sites for each gene in the reference *D. melanogaster* genome (dm3 / BDGP5; the newer version dm6 contains these regions unchanged except the shifted coordinates). We predicted TFBSs in these regions for the transcription factors Bicoid (Bcd), Caudal (Cad), Hunchback (Hb), Giant (Gt), Krüppel (Kr), Knirps (Kni), Tailless (Tll), and Huckebein (Hkb) by using position weight matrices (PWMs) [22]. The PWMs for these TFs were presented in [23] (http://www.autosome.ru/iDMMPMM/), and the thresholds were selected as in [24]. We included a predicted TFBS in the model if it satisfied at least one of the following conditions: (1) the binding site had a high PWM-score and was located in the open chromatin domain according to the DNase I accessibility data [25], or (2) the site overlapped with one of the *cis*-regulatory modules according to RedFly database [26], or (3) the site overlapped with one of the footprint sites [26].

### Gene expression model

We predicted gene expression in the *Drosophila* gap gene network using a hybrid thermodynamic model reported in [6] (denoted as ‘Model 4’ in the cited paper). The model combines the thermodynamic approach for calculating the probability of transcriptional activation of target genes and the reaction-diffusion equations for the spatiotemporal dynamics of gene products, as we describe briefly in what follows and in full details in Additional file 1. The transcriptional activation of a gene is assessed based on the information about the TFBSs in its regulatory sequence and concentrations of the TFs Bcd, Cad, Hb, Gt, Kr, Kni, Tll, and Hkb in nuclei at the A-P axis of the blastoderm-stage embryos during cleavage cycles 13 through 14A. The TFs Bcd, Cad, Tll, and Hkb provide external inputs to the model equations, and the mRNA and protein concentrations for the gap genes *hb*, *Kr*, *gt*, and *kni* are calculated as the model output. Therefore, the model accounts for the regulatory interactions between the four gap genes and the external influence from the other four proteins.

We used values of free parameters in the model that were previously obtained in [6] for *D. melanogaster* reference genome by fitting the model output to the wild type gap gene expression data at cellular resolution (Additional file 1) [27]. Using these parameter values, we calculated the ‘wild-type’ solution *U* ^*p*^(*a*,*i*,*t*), which is the concentration of gene product *p* (*p*=‘mRNA’ or *p*=‘protein’) for gene *a*, nucleus *i*, and time *t*.

### Evolutionary algorithm

We simulated the evolution of 100 haploid, sexually reproducing individuals, holding all parameters, including population size, mutation rate (0.001 per base pair per generation), and selection constant for 3,350 generations. Genotypes are represented as the DNAase accessible regions of four 18kb regulatory sequences (described above) flanking the gap genes *hb*, *Kr*, *gt*, and *kni*. Phenotypes are the spatial and temporal mRNA and protein expression dynamics of the respective *Drosophila* gap gene regulatory network, and are derived by simulating development with an individual’s specific regulatory genotype.

After mutation, phenotypes are computed and then sorted in order of increasing *rms*-scores—a measure of capability to mimic wild-type computational expression patterns. The *rms*-score *F* is calculated as follows:

1 $$ F=\sqrt{\frac{1}{N}\sum_{p,a,i,t}\left(u^{p}(a,i,t)-U^{p}(a,i,t)\right)^{2}},  $$

where *u* ^*p*^(*a*,*i*,*t*) is the model output for the mutated regulatory sequence, and *U* ^*p*^(*a*,*i*,*t*) is the wild-type model output. The summation takes place over all values of the indices for which the experimental data on the wild type gene expression is available. The overall number of the terms in this sum equals to *N*=6800 [6]. From the subpopulation of genomes that produce expression patterns sufficiently greater than or equal to the 20th best individual, two parents are randomly selected with replacement and recombine regulatory sequences with equal probability, until the following generation is populated. Recombination only shuffles disparate regulatory sequences, with no recombination break points within the 18kb sequences. The simulation is seeded with a population of genetically identical wild-type regulatory sequences. The gap gene expression patterns for the 20 best fit individual sequences from the last generation are shown in comparison with the initial patterns in Additional file 2.

### TFBS tracking

The evolutionary trajectories of TFBSs were determined by tracking binding site starting position coordinates. First, we identified TFBSs present in all generations with identical coordinates. For other TFBSs we discriminated between site movement and site loss by comparing neighboring generations. We assumed that a site *S* moved if in the next generation there existed a site for the same TF within the vicinity of *S*. This vicinity of *S* is defined as the sequence, three times the site length, flanking and containing *S*. If there were no TFBSs for the same TF in this vicinity, *S* is considered to have disappeared. In addition to site deaths, site births occur when a TFBS appears in a generation where there were no sites within its vicinity previously. A birth event is considered to be a rebirth if the new site is located in the above-stated vicinity of the wild type coordinates of *S*. The algorithm stores the trajectories of all TFBSs for a randomly chosen individual in each generation. We used TFBS tracking for individuals randomly chosen from the 20 most fit individuals from each generation. For TFBSs other than the tracked ones, the averaging of the binding energy *E* and other quantities is taken across the 20 most fit individuals from each generation. This averaging is justified since the sequences do not show significant multimodality in *E* distributions (Additional file 2).

## Results

**Fig. 1:**
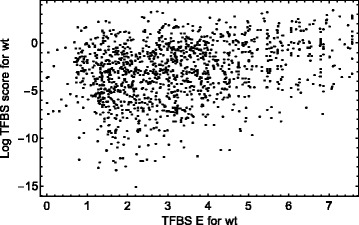
Correlation between the binding energy *E* and the log-transformed regulatory *rms*-score of TFBSs from the gap gene regulatory regions. The scatterplot is computed for the wild-type conditions, i.e. for the initial regulatory sequence

We estimate the phenotypic importance of an individual TFBS in the initial (wild-type) regulatory sequence by calculating the *rms*-difference between the model wild-type expression patterns, with and without a particular TFBS. We call this difference the regulatory *rms*-score of the binding site, and larger values of this score correspond to a stronger influence of the TFBS on expression, and vice versa [6]. Our model reveals only a small correlation between the binding affinity of a TFBS and its functional importance assessed via its *rms*-score (Fig. 1; the Pearson correlation coefficient CC=0.30). In what follows, we investigate how this small correlation leads to the specific variability of the binding energy profile during the simulated evolution of the regulatory sequences.

### Sequence simplification

**Fig. 2:**
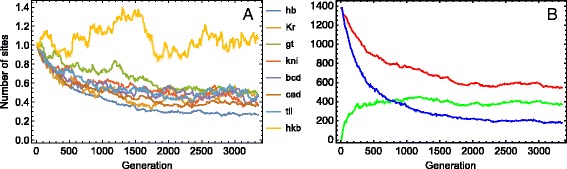
Dynamics of the number of TFBSs. **a** The generation average number of all binding sites for each TF is shown normalized by the initial value of this number. These are the generation average initial/final numbers of TFBSs for each TF: 507/135 (Hb), 122/57 (Kr), 125/60 (Gt), 116/43 (Kni), 150/69 (Bcd), 184/66 (Cad), 121/58 (Tll), 55/58 (Hkb). **b** The dynamics for all TFBSs (*red*), the initial set of sites (*blue*), and sites born during the simulation (*green*). The *red* and *green curves* were obtained by averaging the corresponding numbers of sites across the 20 best individuals in the population, while the *blue curve* stems from the site trajectories tracked across a randomly chosen sequence from the 20 best fit individuals from each generation

Evolution was simulated under an elevated mutational pressure (mutation rate *μ*=0.001) leading to a decrease in regulatory sequence complexity, as there was a significant drop in the total number of TFBSs for almost all TFs (Fig. 2 a). The dynamics of the total number of sites is balanced by a steep decrease in the the number of initial sites and a saturating increase of the number of sites born throughout evolution (Fig. 2 b). The sequence reacts to the elevated mutation rate by changing its TFBS composition, with new sites surviving twice as long as the initial sites. The final quasi-steady numbers of TFBS for each TF is a result of mutation selection balance.

The TFBS disappearance rate also depends on TFBS motify redundancy, determined by the number of sequence motify of similar affinity and nucleotide sequence available to a TF. Such a TF should have a large number of self-overlapping sites and, as a consequence, a higher probability of site loss because a single substitution in the overlapping region is able to destroy multiple sites simultaneously. We can use the fraction of self-overlapping events in the initial set of TFBSs as a measure of such redundancy. When this fraction is large, the double site loss due to mutation in the overlapping region is expected to be significant, leading to a decrease in this fraction as the total number of sites decreases. On the other hand, when the fraction of self-overlaps is initially small, single sites will disappear predominantly with time, hence, the fraction of self-overlaps will increase or stay constant as the total number of sites decreases.

**Fig. 3:**
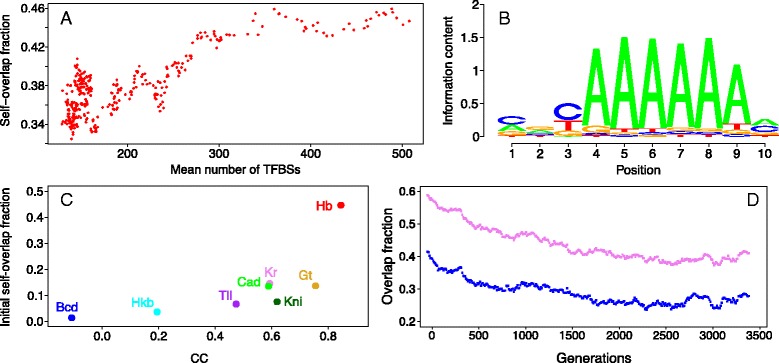
Analysis of TFBS overlapping events. **a** The generation average number of self-overlapping events for Hb TFBSs vs. the generation average total number of TFBSs for the TF. The number of self-overlaps in a generation is shown as a fraction of the average total number of Hb sites in this generation. **b** The sequence logo of the consensus TFBS motif for Hb. The analogs of (**a**) and (**b**) for other TFs are shown in Additional file 2. **c** The number of TFBS self-overlaps for each TF in the initial regulatory sequence, as a fraction of the initial number of sites for each TF, vs. the correlation coefficients for the TF-specific analogs of the scatterplot shown in (**a**). **d** The dynamics of the population average number of overlapping events between all TFBSs (*magenta*) and between all but Hb sites (*blue*). The number of overlaps is computed as a fraction of the population average total number of sites under consideration

We observe a relationship between Hb TFBS motif redundancy and Hb TFBS loss: there is a high positive correlation between the fraction of self-overlaps and the total number of TFBSs (Fig. 3 a, c). This is consistent with the fact that Hb TFBSs exhibit the steepest decline in Fig. 2 a. Initially Hb had the largest number of binding sites because it binds to self-repeating polyA and polyT segments (Fig. 3 b) and, thus, has many self-overlapping sites. This effect is present in other TFs but to a lesser degree (Fig. 3 c), i.e. the larger initial fraction of self-overlaps, the more sites are lost due to the double-site mutations.

The fraction of overlapping events among all TFBSs (not only for the same TF) also decrease with time (Fig. 3 d), showing that the same logic holds for heterotypic overlapping. The essential part in this fraction is due to Hb sites, however excluding these sites does not change the reduction trend.

### Variability of binding energy for different sets of sites and sequence annotations

**Fig. 4:**
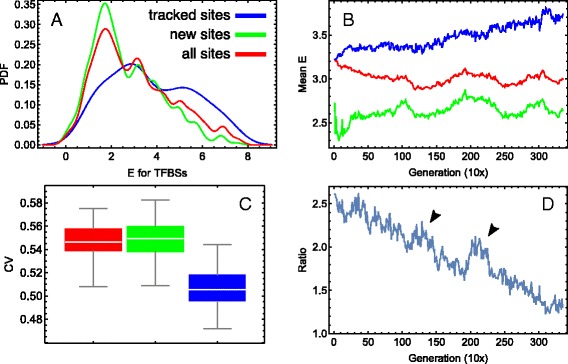
Binding energy variability for tracked and new TFBSs. **a** Probability density functions (PDF) for the last generation distribution of the binding energy *E* for all TFBSs (*red*), new TFBSs (*green*), and sites tracked from the initial set of sites (*blue*). **b** Dynamics of the mean energy for the same three sets of sites as in (**a**), with the same colour code. For all and new sites, the energy was averaged across the 20 best individuals, and for the tracked sites, across the sites of a randomly chosen sequence from the 20 best fit individuals. **c** Temporal distributions of the coefficient of variation (CV =SD/mean) of *E* for the same three sets of sites as in (**a**) and (**b**), with the same colour code. The CV values correspond to the *E* variation within the population in a given generation, and the plotted distributions comprise CV values for all generations. The first 100 generations were not taken into account in the distributions due to a small number of new sites in that period (see Fig. 2 b). **d** Dynamics of the low energy sites number to the high energy sites number ratio for the tracked TFBSs. The sites were classified to the low and high energy sets using *E*=4 as the threshold. The *arrows mark* two stochastic bursts of the amount of low energy sites during the evolution

We follow individual TFBSs (referred to as “tracked sites”), present in high fitness individuals, through the course of the simulation. Tracked sites’ binding energy distributions differ significantly from newly appearing sites’ distributions, and are also distinct from the total distribution (Fig. 4 a–c). The tracked sites exhibit an excess of higher energy sites compared to the new sites (Fig. 4 a). As the new sites eventually form the majority (Fig. 2 b), the total *E* distribution follows that for the new sites. Mean energy for the tracked sites increases with time, whereas the new sites’ mean energies oscillate stochastically around a quasi-steady level (Fig. 4 b). The tracked sites also have lower energy variability compared with the majority of sites (Fig. 4 c). These results suggest that, despite being in an environment of vast sequence variability, the initial TFBSs do not dissolve with time in newly born sites and keep robust function as a whole.

**Fig. 5:**
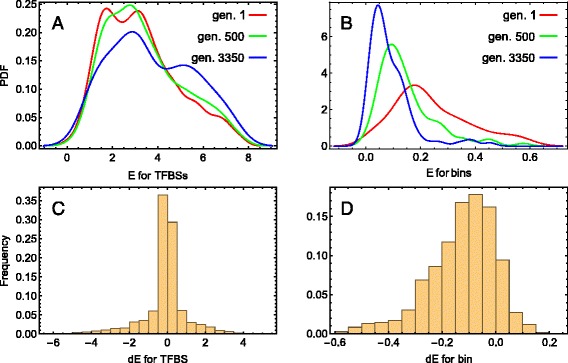
Binding energy variability for the tracked TFBSs and sequence segments. Results for TFBSs are shown in (**a**, **c**), and for the sequence segments (bins) in (**b**, **d**). **a**, **b** The probability density functions (PDFs) for the within-generation distribution of the binding energy *E* are shown for three different generations: the first, mid, and last ones. **c**, **d** Distributions of *d* *E*=*E*−*E* ^wt^ values for all TFBSs (or bins) and all generations

The within-generation distributions of *E* for the tracked TFBSs are indistinguishable between the first and mid generations (*p*=0.32 based on the bootstrapped Kolmogorov–Smirnov (KS) test), while for the last generation the distribution shows a small bias towards larger *E* values (*p*=0.02; Fig. 5 a). The statistical difference with the first generation becomes persistent only after approximately the 2500th generation and demonstrates rather damping oscillations before that (Additional file 2). These oscillations correspond to stochastic events during evolution associated with the appearance of many low energy sites over a short time period (Fig. 4 d), in opposition to the main trend of such site loss within the tracked sites.

To further examine sequence simplification, we consider a coarser grained sequence annotation and compare its energy profile properties with TFBS energies. We arbitrarily break each gap gene regulatory sequence into 180 nucleotide bins (or segments), resulting in 104 bins for the four genes. For each segment, we define a binding energy (per bp) *E*_bin_ as the sum of the TFBS binding energies within each segment, normalize by segment length, and average across individuals in a generation. Therefore, *E*_bin_ represents the ‘binding capacity’ of a segment: the more high affinity sites it contains, the larger *E*_bin_ it has.

This annotation leads to a qualitatively different picture of binding affinity variation. While the tracked TFBSs evolve towards a larger average energy (Figs. 4 b, 5 a), the dynamics of the *E*_bin_ distribution exhibits a shift towards smaller mean values and smaller variances (Fig. 5 b). This shift is related to the loss of TFBSs, as fewer sites in a bin lead to smaller energy weights of nucleotides within each bin. The *E*_bin_ distribution is first distinguished from the distribution at the initial generation at approximately generation 220. Calculating the difference *d* *E*=*E*−*E* ^wt^ between the binding energy during evolution with its wild-type (initial) value *E* ^wt^ for the sites and bins, we see that, in contrast to the individual TFBSs, the difference ${dE}_{\text {bin}}=E_{\text {bin}}-E_{\text {bin}}^{\text {wt}}$ for the bins has a distribution shifted towards negative values (Fig. 5 c, d). Therefore, the sequence simplification due to the elevated mutational pressure leads to opposite trends at the level of the binding energy for different sequence annotations.

### Correlations with *rms*-scores of individuals

**Fig. 6:**
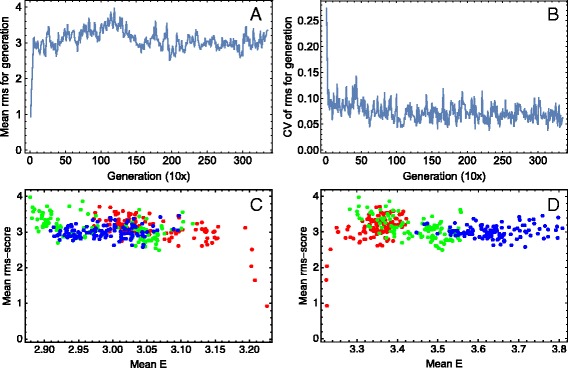
The *rms*-score dynamics and correlation with binding energy. **a**, **b** Dynamics of the generation mean (**a**) and coefficient of variation (**b**) for individual *rms*-scores. **c** Correlation (across generations) between the generation mean *rms*-score of individuals and the generation mean *E* for all TFBSs. The *red* points correspond to the first 1100 generations, the green ones to the next 1100 generations, and the *blue* points to the last 1150 generations. **d** The same as in (**c**) but for the tracked TFBSs, with the same colour code. The mean *E* in this case is computed across all tracked sites in the best fitted individual in a given generation

The per generation mean and normalized SD of individuals’ *rms*-scores saturates rapidly with a slight decline of the CV through time (Fig. 6 a, b). We may consider these dynamics as a stochastic fluctuation of the system around a quasi-steady state of constant fitness. We also see this fast saturation in the figure of correlation between mean *rms*-score and mean energy for various sets of TFBSs (Fig. 6 c, d), where an *rms*-score increase is accompanied with a decrease (increase) of the mean *E* for all (tracked) sites. These figures also show that, in the quasi-steady fitness regime, there are no significant correlations between fitness of the individual and the mean energy. The same is true for correlation between the CVs for *E* and *rms*-score (Additional file 2).

### Mutual correlations between sets of sites and sequence segments

The rapid change of binding energies throughout evolution inevitably leads to some general patterns of correlations between temporal profiles of *E* for distinct TFBSs and their groups. One such correlation pattern corresponds to the fact that low affinity sites are maintained through time only if their binding energies increase, whereas high affinity sites are more likely to experience an energy decrease. As a consequence, we may expect higher positive correlations within the groups of low and high affinity binding sites.

**Fig. 7:**
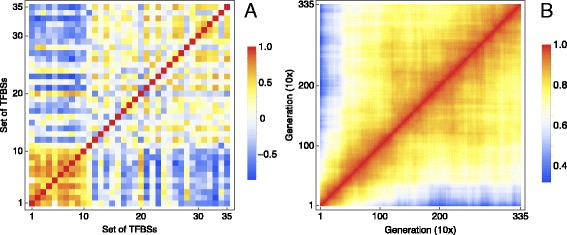
Correlation matrices for *E* profiles for TFBS clusters and sequence bins. *Left*: Correlations of the temporal *E* profiles for the sets of TFBSs of varying affinity. Set #1 (#35) corresponds to the tracked sites initially having the smallest (largest) *E*. *Right*: Correlations of the sequence *E*_bin_ profiles for all pairs of generations. See the text for full details of these figures

In order to look at such correlations, we arranged the tracked TFBSs in order of their, increasing, initial (wild-type) binding energies and partitioned this list of sites into non-overlapping 35 sets. Then for each generation, we averaged energy inside each site set. Finally, we correlated the samples of averaged energies for all generations for any pair of the site sets (Fig. 7 a). The clusters of positive correlations in the lower left and the upper right corners of this figure correspond to the described correlation pattern for the low and high affinity sites, respectively. This general correlation pattern may hide functional correlations between TFBSs at the level of binding energy, but it can be used as a background model for finding such functional correlations by statistical methods.

We can also use the correlation matrices to answer the question about how fast the binding energy distribution in the sequence changes throughout evolutionary time. In this case, we calculated correlations between the distribution of *E*_bin_ in the sequence for a given generation with the same distributions for all other generations (Fig. 7 b). The narrow regions of small correlations in this figure illustrates the two properties of the evolutionary simulation: the rapid early change of the energy profile and the later saturation, making more distant generations hardly distinguishable.

### Core TFBSs and their vicinity

**Fig. 8:**
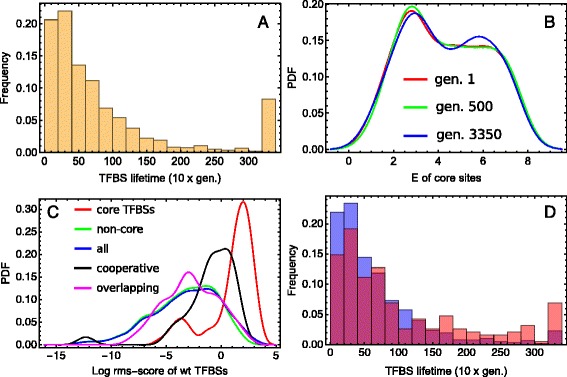
Statistics for the core TFBSs and their vicinity. **a** Distribution of lifetimes for the tracked TFBSs. **b** The within-generation distributions of the binding energy *E* of 85 core TFBSs shown for three different generations (analog of Fig. 5 a for the core sites). **c** Distribution of the regulatory *rms*-scores of TFBSs in the model (the strength of influence that a site exerts on gene expression) for the initial wild-type conditions, for five sets of TFBSs: core sites, non-core sites, all sites, sites cooperatively interacting with the core sites, and non-core sites overlapping with the core sites. **d** Distributions of lifetime for the neighbouring TFBSs (*red*) and for all except core binding sites (*blue*). The difference between the red and blue distributions are statistically significant under 5 percent level based on the bootstrapped KS test

The extensive loss of tracked TFBSs through time leads to a broad longevity distribution, with a mode corresponding to longer-living TFBSs (Fig. 8 a). We checked whether TFBS lifespans change during evolutionary time, i.e. whether later born sites display increased or decreased longevity compared with earlier born ones. We compared the lifetime distribution of the tracked binding sites for sites that were born and then died within the first 500 generations with those from the last 500 generations, and did not find a statistical difference between these distributions (Additional file 2). However, if we take into account the initial sites that die in the first 500 generations, the average lifespan for the group of early sites becomes larger than that for the later sites.

We extracted 85 TFBSs that remained in the population for the entire simulation (‘core binding sites’) and investigated their behavior (the placement of these sites in the sequence is illustrated in the genome-browser like figures in Additional file 2). The analog of Fig. 5 a implies that the binding energy distribution across core sites is highly conserved during evolution (Fig. 8 b: The appearance of the high-affinity mode in the last generation is not statistically significant). The core sites are clearly segregated from other tracked TFBSs with respect to their wild-type regulatory *rms*-scores (Fig. 8 c). Functionally important TFBS are typically core sites as well.

We also studied how a TFBS’s local sequence environment influences its evolutionary dynamics. We considered two types of the tracked non-core TFBSs in the vicinity of the core sites. The first type are sites located no farther than 50 bps from at least one core site of the same TF, being thus under conditions of the cooperative interactions with such core sites (Additional file 1). There are 61 such cooperative sites in total. The second type are TFBSs overlapping with at least one core site (and not present in the set of cooperative sites); there are 127 such sites. We call the joint set of TFBSs of both types neighboring sites.

Just like the core sites, the neighboring sites tend to be maintained for longer periods of time (Fig. 8 d), suggesting that sites within the vicinity of core TFBSs are more highy conserved during evolution. Interestingly, the two types of the neighboring sites possess different distributions of the wild-type *rms*-scores (Fig. 8 c). The cooperative sites are clearly distinguishable from the majority of sites and hold a rather intermediate position between the full distribution and the distribution for the core sites. The sites overlapping with the core ones are indistinguishable from the majority of sites, showing no essential influence on gene expression for the wild-type conditions. This difference can be explained by different types of influences on the core sites. While the cooperative sites actively influence the core TFBSs, the sites overlapping with the core TFBSs may live longer due to negative selection of mutations in the overlapping region with the core site. Therefore the higher retention rate for the sites overlapping with the core TFBSs can be interpreted as an example of a selective sweep.

The bimodality of the *rms*-score distribution of the core sites is also related with the overlapping between sites. The left peak of the red curve in Fig. 8 c is formed by the core sites that overlap with other core sites (Addition file 2). As these sites have a small impact on gene expression (for the initial sequence), we may suggest that their ultimate longevity is explained solely because they overlap with core sites that have a stronger influence on expression.

## Discussion

In this study, we have provided an example of regulatory sequence change through evolutionary time. We pondered that given the large set of phenotypically and functionally equivalent regulatory schemes (when varying TFBS quantity, affinities, availability, etc.) that only a subset of these will be explored during evolution, and are contingent upon specific acting population, molecular, and historical forces. Previously, it was shown that high quantity TFBS sequences make up a relatively larger portion of acceptable sequence space and are consequently more likely to be frequented during evolution [10]. Mutational pressure seems to bias the evolutionary search of suitable regulatory sequences towards fewer TFBSs.

Under significant mutational pressure, our simulations demonstrate that the cumulative number of phenotypically important TFBSs diminishes in concert with an increase in average binding site affinities. This is likely a direct consequence of the mutation rate, as having fewer TFBS, and thus fewer functionally relevant nucleotides, minimizes the mutational target of the genome. Despite this constraint, the *Drosophila* gap gene network ably compensates by tweaking the binding energies of the remaining TFBSs. The observed change in low- and high-affinity binding sites is likely a result of compensatory actions. The tradeoff between TFBS frequency and affinity seems to be a general feature of regulatory networks [28]. This may also be consistent with claims that much of regulatory sequence organisation is predominantly shaped by neutral forces [20].

In this simulation, we do not only observe sequence drift, but we also notice qualitative changes in higher level descriptors of the regulatory network (such as binding energy). One might reasonably expect that wild-type regulatory sequences and higher-level network descriptors would already be optimized and at equilibrium, and that any of the observed qualitative changes here are a consequence of specific experimental conditions. This is seemingly a parsimonious explanation of our results and further reveals the specific influence of the mutation rate on the equilibrium values of higher-level network descriptors. If fewer TFBSs than are present in the wild-type sequence can achieve nearly identical expression patterns, is there a selective advantage to the wild-type regulatory strategy, or is it merely a consequence of neutral processes [10, 20, 21]? Our results showing slightly larger deviations of expression in derived populations could hint at the involvement of selective forces (overwhelmed in the present study). It is also important to note that our methodology focuses solely on several minutes of early development and our artificial version of selection is restricted to assessing brief spatiotemporal expression patterning, neglecting any extraneous potential functionality. Consequentially, a population at or near selective equillibrium, experimentally subjected to only a subset of selective criteria, may incorrectly appear to be unneccessarily complex.

One non-neutral possibility is that the more TFBSs involved in regulation, the more fine-tuned expression patterns can be. However, the increased control provided by additional TFBSs may diminish as a function of TFBS quantity. As such, the benefits provided by each additional fine-tuning site will eventually be outweighed by the cost of maintaining a larger mutational target. Although beyond the scope of the present study, these trade-offs, if necessary, may be better understood and predicted within the context of population genetic modeling. Future research can add mathematical clarity to the above speculations and further elaborate the connections among specific population parameters and network descriptor equilibriums. Originally developed within the context of RNA evolution and mutation, mathematical models that examine the tradeoffs among replicative fidelity of a sequence, sequence length, and mutation rates, may provide further insight [29].

Core TFBSs, TFBSs that remain throughout the entire evolutionary history of the simulation, are identified and shown to be extremely important towards maintaining wild-type expression patterns. In contrast, many sites with smaller phenotypic effects turnover often, and are suggested candidates responsible for observed sequence drift in actual biological populations. Another result of this simulation, consistent with previous work [5, 6], is the fairly weak correlation between TFBS energy and the impact of its removal on the entire system’s dynamics. This further illustrates the necessity of realistic computational genotype-phenotype maps to assigning evolutionary functionality. The selective importance of many TFBSs can only be deeply understood within the context of the system it operates in, and not solely from local measurements.

We also note that TFBSs within close proximity of core sites are retained at a higher rate than other non-core TFBSs, despite sharing a similar perturbation distribution with other non-core, non-proximal binding sites. In accordance with our expectations of the influence of mutational pressure, this pattern may be a consequence of these non-core TFBSs sharing nucleotides with neighboring core TFBSs, and thus containing fewer mutable nucleotides. Others also observed increased longevity of overlapping binding sites [21]. Additionally, close core proximity TFBSs may be favorable due to cooperative interactions of these sites with the core sites.

The presented results have some limitations. We analyzed the results of a single evolutionary simulation with a fixed mutation rate. New simulations with varying parameter schemes are likely to clarify and refine our understanding of sequence evolution. In particular, the core TFBSs and other aspects may be different in other simulations, as evolution is a stochastic process. Despite the complexity of our model and its detailed focus on molecular mechanisms, it has mechanistic limitations as well. Only a subset of the known mechanistic details in regulation are simulated and, in particular, the 3D genome organisation, is presently neglected. Models that include these detials might disagree on the position of DNA and on the regulatory role of certain TFBSs, and potentially lead to different observations. Overcoming these modelling limitations is a desirable condition for future evolutionary studies.

## Conclusions

Our simulations of genetic regulatory network evolution suggest that in response to elevated mutational pressure, the size of the functional regulatory sequence decreases to minimize risk. To compensate for this organisational constraint, TFBSs with, on average, stronger binding affinities are selectively maintained. The small correlation between TFBS affinity and functional importance means that gene network evolution tends towards sequence organisations having many weak TFBSs working in concert. This can make conclusions solely based on the analysis of the binding affinity landscape vague and incomplete. However, we show that core TFBSs, which form the regulatory backbone of the network, are highly conserved and can be identified at the level of binding energy dynamics. TFBSs that interact with these core binding sites also exhibit increased longevity. Despite the present study’s focus on a specific system and parameter regime, its results will likely be relevant to more general studies of the TFBS evolutionary landscape, and its conclusions useful to systems lacking genotype-phenotype maps.

## Additional files

Additional file 1 Model equations and parameter values. (PDF 141 kb)

Additional file 2 Supplementary figures. (PDF 2437 kb)
