# Supplementary material for: In silico evolution of the Drosophila gap gene regulatory sequence under elevated mutational pressure
Source: BMC Evol Biol. 2017 Feb 7;17(Suppl 1):4. doi: 10.1186/s12862-016-0866-y (PMC5333172; doi:10.1186/s12862-016-0866-y)
Supplement: Additional file 1 — Model equations and parameter values. (PDF 141 kb) [file 12862_2016_866_MOESM1_ESM.pdf]

Additional file 1: Gene expression model

In this text, we present details of Model 4 from (Kozlov et al., 2015), which was used for the evolutionary simulations. This model describes the expression of gap genes *hunchback* (*hb*), *Kruppel* (*Kr*), *giant* (*gt*), and *knirps* (*kni*) during the blastoderm stage of *Drosophila melanogaster* development. These genes participate in the formation of the segmentation prepattern underlying the body plan of the fruit fly (Jaeger, 2011). The embryo consists of nuclei undergoing a series of synchronous divisions during this stage. We consider a time lapse from the 13th nuclear cleavage cycle to the end of the cycle 14A, and nuclei from the anterior-posterior (A-P) axis of the embryo ranging from 35% to 92% of the embryo length.

The expression dynamics of the gap gene network inside each nucleus is according to the following reaction-diffusion equations (Kozlov et al., 2015, 2014):

$$\frac{du_i^a}{dt} = R_u^a E_i^a(t) - \lambda_u^a u_i^a + D_u^a(n)[(u_{i-1}^a - u_i^a) + (u_{i+1}^a - u_i^a)], \tag{1}$$

$$\frac{dv_i^a}{dt} = R_v^a u_i^a(t - \tau^a) - \lambda_v^a v_i^a + D_v^a(n)[(v_{i-1}^a - v_i^a) + (v_{i+1}^a - v_i^a)], \tag{2}$$

where u_i^a and v_i^a are mRNA and protein concentrations, respectively, of the products of gene a in nucleus i, R_v^a and R_u^a are maximum synthesis rates, D_v^a and D_u^a are diffusion coefficients, λ_v^a and λ_u^a are decay rates, n is the cleavage cycle number, and the τ^a are time delay parameters accounting for the delay between transcription initiation and protein appearance. These equations implement three processes for the gene products (from left to right on the right hand side of the equations): synthesis, decay, and diffusion (nucleus-to-nucleus transport). The mRNA synthesis term depends on the probability of transcriptional activation $E_i^a(t)$ of gene a in nucleus i at time t. The protein synthesis is proportional to the mRNA concentration. The list of transcription factor (TFs) regulating the activity of the four gap genes include their own proteins (Hb, Kr, Gt, and Kni) and the proteins of four external regulators: Bicoid (Bcd), Caudal (Cad), Tailless (Tll), and Huckebein (Hkb).

The probability of transcriptional activation $E_i^a(t)$ is interpreted as being equivalent to the fractional occupancy of the promoter by the basal transcriptional machinery (BTM) and calculated using a thermodynamic approach in the form proposed by He et al. (2010) and further modified in our papers (Kozlov et al., 2014, 2015). In what follows, we describe derivation of this probability omitting the indices a and i and time variable t for brevity.

The statistical thermodynamics approach counts all possible configurations of the complex that consists of the regulatory region of a gene together with its basal promoter. We assign the 'ON' state to those configurations of the complex in which the BTM is bound to the promoter, and the 'OFF' state to the configurations in which the promoter is unoccupied. A configuration of the regulatory region is indexed by a vector $\sigma = \{\sigma(s)\}$ containing the information about the occupation status of the transcription factor binding sites (TFBSs) in the region: $\sigma(s) = 0$ if TFBS s is free and $\sigma(s) = 1$ if this site is occupied. The occupancy of site s is characterized by the statistical weight q_s:

$$q_s = Kv \exp(\Delta P_s), \tag{3}$$

where K is the association constant for a strongest TFBS (one constant per TF), v is the protein concentration for the TF binding to site s, and ΔP_s is the difference between a PWM (positional weight matrix) score of site s and the score of the strongest site.

Given the statistical weights of all TFBSs in a configuration σ of the regulatory region, we calculate the statistical weight W_σ of this configuration as follows:

$$W_\sigma = \prod_s (C_s q_s)^{\sigma(s)}, \tag{4}$$

where the exponentiation to the power of $\sigma(s)$ guarantees that only the weights q_s of occupied TFBSs are present in W_σ. The parameter C_s accounts for two possible types of local interactions between TFs bound to site s and to other sites in a given sequence range: cooperative binding and short-range repression. If site s binds TF cooperatively with neighboring sites j of the same TF, we have

$$C_s = \prod_j \omega^{\sigma(j)}, \tag{5}$$

where $\omega > 1$ is the cooperativity parameter (one parameter per TF). The short-range repression mechanism provides the inhibition of the target gene by inactivating TFBSs for activators (forbidding the binding to these sites) in the vicinity of TFBSs occupied by repressor TFs. According to this mechanism, a TFBS occupied by a repressor can be in two states: effective and ineffective. It is assumed that the DNA in the vicinity of the site in the effective occupied state changes its local conformation making this vicinity inaccessible for binding, while the ineffective state does not influence the local DNA. If site s binds TF-repressor and is in the ineffective state in configuration σ, we write $C_s = 1$. For the effective state, we have $C_s = \beta$, where the parameter β quantifies the repression efficiency (one parameter per TF-repressor and target gene). The cooperativity and repression parameters are multiplied in C_s if the corresponding mechanisms coexist for a given site and configuration.

A configuration σ of the regulatory region with the weight W_σ may lead to either free basal promoter or the promoter occupied by the BTM, i.e. to either OFF or ON state of the complex, respectively. The statistical weight of the ON state equals to $W_\sigma Q_\sigma$, where Q_σ accounts for the interaction with the BTM of activator TFs bound in the regulatory region. Under the limited contact hypothesis, we assume that not more than N activator TFs can interact simultaneously, either directly or via adaptor factors, with the BTM (He et al., 2010). Therefore, we have for Q_σ:

$$Q_\sigma = \sum_{k=1}^{N} \sum_{i_1,\dots,i_k} \alpha(s_{i_1}) \cdots \alpha(s_{i_k}), \tag{6}$$

where the summation is taken over all different k-length ($k \leq N$) combinations of activator TFBSs $(s_{i_1}, \dots, s_{i_k})$ occupied in the configuration σ, and $\alpha(s) = \alpha^{\mathrm{TF}(s)}$ is the activation efficiency parameter for each activator TF (one constant for each activator TF and target gene). The statistical weight of the OFF state of the complex equals to W_σ, i.e. the relative weight of the empty promoter is set to unity.

Finally, the probability of transcriptional activation is calculated as a fractional occupancy of the basal promoter in the complex with the regulatory region:

$$E = \frac{Z_{ON}}{Z_{ON} + Z_{OFF}}, \quad Z_{OFF} = \sum_\sigma W_\sigma, \quad Z_{ON} = \sum_\sigma W_\sigma Q_\sigma, \tag{7}$$

where Z_{ON} and Z_{OFF} sum up statistical weights of all possible states of the complex with the BTM bound and not bound to the basal promoter, respectively (He et al., 2010; Kozlov et al., 2014, 2015). As the TF protein concentrations vary for genes a, nuclei i, and times t, i.e. $v = v_i^a(t)$ in eq. (3), we have the same dependence for E: $E = E_i^a(t)$.

The model also implements a possible dual regulatory action of TF a on target gene b, when the type of action (either activation or repression) depends on the TF concentration v_i^a: it is activation for small concentrations and repression for large ones. In this case, we replace a single parameter quantifying the regulatory action strength (either activation efficiency α or repression efficiency β) by three parameters: a threshold concentration V^a, an activation strength α for the case when $v_i^a \leq V^a$, and a repression strength β for the case when $v_i^a > V^a$. This dual regulatory interactions are assumed for the mutual regulation of gap genes *hb* and *Kr* (Kozlov et al., 2015).

The parameter values (Table S1) were obtained by fitting the model output to the wild type gap gene expression data at cellular resolution (Pisarev et al., 2009), as described by Kozlov et al. (2015). We used methods of cross-validation analysis, fitting to nonsense data, and local identifiability analysis to show previously that the model is relatively stable to overfitting (Kozlov et al., 2014). The model predictions for parameter values from Table S1 were successfully tested on the expression data for a number of experimentally characterized genetic constructs (Kozlov et al., 2015).

References

Xin He, Md Abul Hassan Samee, C Blatti, and Saurabh Sinha. Thermodynamics-based models of transcriptional regulation by enhancers: the roles of synergistic activation, cooperative binding and short-range repression. *PLoS computational biology*, 6(9):e1000935, 2010.

Johannes Jaeger. The gap gene network. *Cellular and molecular life sciences : CMLS*, 68(2): 243–274, January 2011.

K N Kozlov, Vitaly V Gursky, Ivan Kulakovskiy, and Maria G Samsonova. Sequence-based model of gap gene regulatory network. *BMC genomics*, 15(Suppl 12):S6, 2014.

K N Kozlov, Vitaly V Gursky, Ivan V Kulakovskiy, Arina Dymova, and Maria G Samsonova. Analysis of functional importance of binding sites in the Drosophila gap gene network model. *BMC genomics*, 16(Suppl 13):S7, December 2015.

Andrei Pisarev, Ekaterina Poustelnikova, Maria G Samsonova, and John Reinitz. FlyEx, the quantitative atlas on segmentation gene expression at cellular resolution. *Nucleic Acids Research*, 37(Database issue):D560–6, January 2009.

Table S1. Parameter values in the model.

	Hb	Kr	Gt	Kni	Bcd	Cad	Tll	Hkb
α/β for *hb*	426	6525/126	518	8609	635	290	1068	266
α/β for *Kr*	2148/200	281	697	353	638	465	1374	305
α/β for *gt*	1978	3926	4840	469	15	97	565	2997
α/β for *kni*	2109	572	460	205	12	1020	1516	1740
K^a	0.0001	0.0001	0.0475	0.0471	0.0125	0.0121	0.0498	0.0120
ω^a	1	1.1	1.5	5.2	1	1	4.7	1
τ^a	3.5	1.3	7.3	1.0	–	–	–	–
R_u^a	0.0760	0.0954	0.0951	0.1050	–	–	–	–
R_v^a	11.10	20.55	20.19	9.70	–	–	–	–
λ_u^a	1.14	1.38	3.07	1	–	–	–	–
λ_v^a	1.55	16.61	8.93	8.28	–	–	–	–

The first four rows contain the activation efficiency parameters (α, shown in red) and the repression efficiency parameters (β, shown in blue) for each TF (in columns) and target gene (in rows). Each TF except Hb and Kr can be either activator or repressor for a given target gene. The regulatory interactions between genes *hb* and *Kr* are of the dual type, so the action of Hb on *Kr* and Kr on *hb* is characterized by two action strength parameters (β/α). The protein concentration thresholds delimiting the activating and repressing actions (see the text) are $V^{\mathrm{Kr}} = 13.40$ and $V^{\mathrm{Hb}} = 52.27$ (a.u.) for TFs Kr and Hb, respectively. K^a is the affinity constant for the strongest binding site of each TF. ω^a is the cooperativity parameter. τ^a is the delay time (in minutes). R_u^a and R_v^a are the maximal synthesis rates for mRNA and protein, respectively. λ_u^a and λ_v^a are log2/halftime (in minutes^{-1}) for mRNA and protein, respectively. The parameters from the last five rows are only for the four gap genes. The maximal number of activator molecules allowed to interact with the BTM: $N = 3$. The repression range (in basepairs) for the short-range repression mechanism: $d = 198$. An additional parameter $q_{\mathrm{BTM}} = 4.5 \times 10^{-6}$ is a factor by which Z_{ON} from Eq. (7) is multiplied. This parameter represents the basal level of the BTM-promoter interaction, so the statistical weights corresponding to all other ON-states are counted from that level (He et al., 2010). All parameter values in the table and listed above were found by the differential evolution optimization method minimizing the difference between the model output and the wild type gap gene expression data. The following diffusion parameters from the model equations were fixed during optimization: $D_u^a = 0.05$ and $D_v^a = 0.005$ for each gene a; these are values for the cleavage cycle 14A, and they are divided by 4 for the previous cycle since the internuclear distance in cycle 13 is twice the distance in cycle 14A.
