# Supplementary material for: In silico evolution of the Drosophila gap gene regulatory sequence under elevated mutational pressure
Source: BMC Evol Biol. 2017 Feb 7;17(Suppl 1):4. doi: 10.1186/s12862-016-0866-y (PMC5333172; doi:10.1186/s12862-016-0866-y)
Supplement: Additional file 2 — Supplementary figures. (PDF 2437 kb) [file 12862_2016_866_MOESM2_ESM.pdf]

Supplementary figures

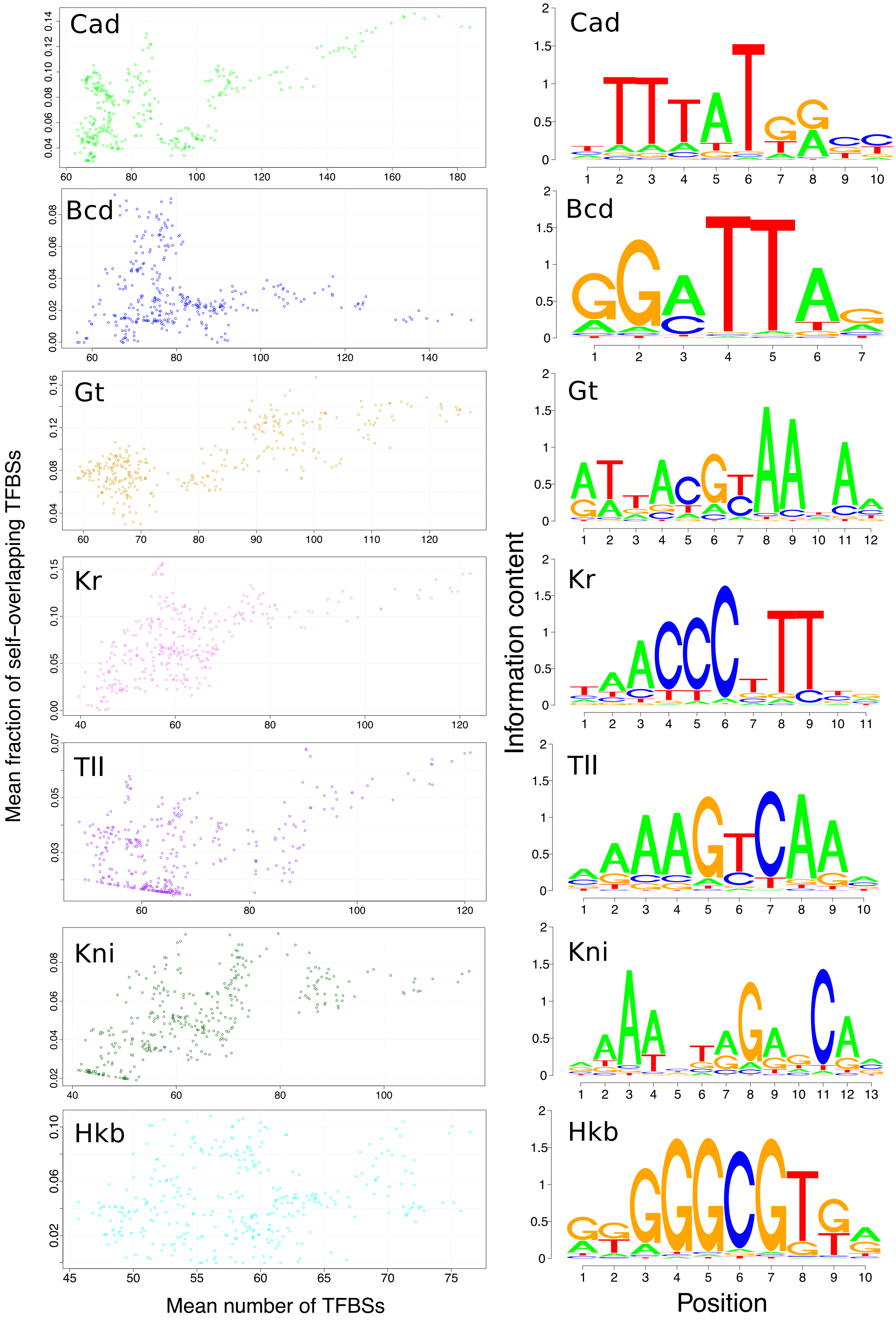

Figure S1. Analogs of panel (A) (left column) and panel (B) (right column) of Figure 3 from the main text, for all TFs except Hb.

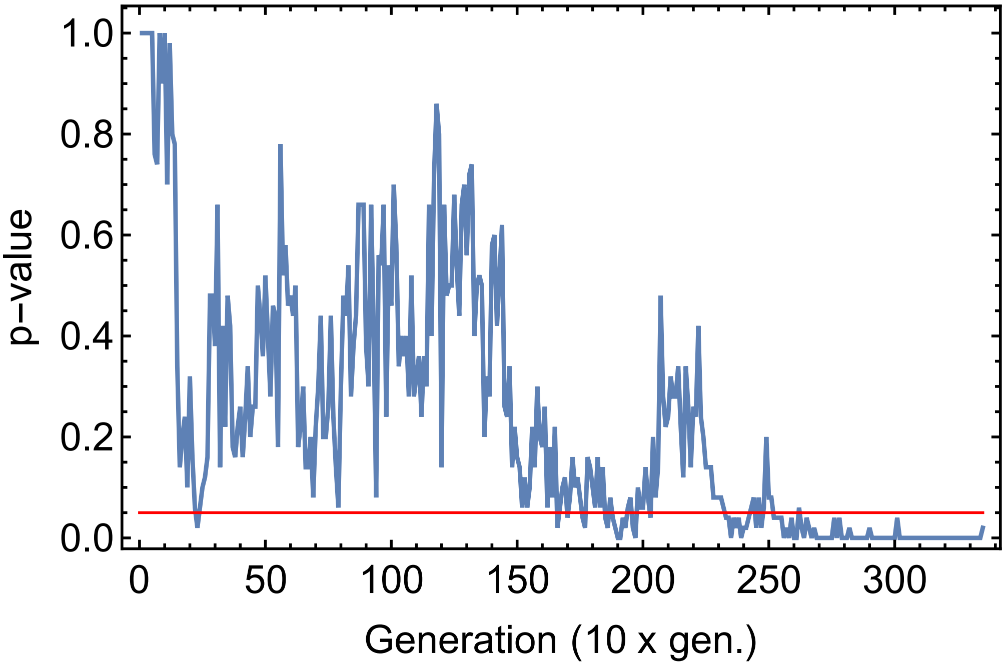

Figure S2. Figure with the dynamics of the p-value for the *E* distribution difference from the first generation (for the tracked TFBSs).

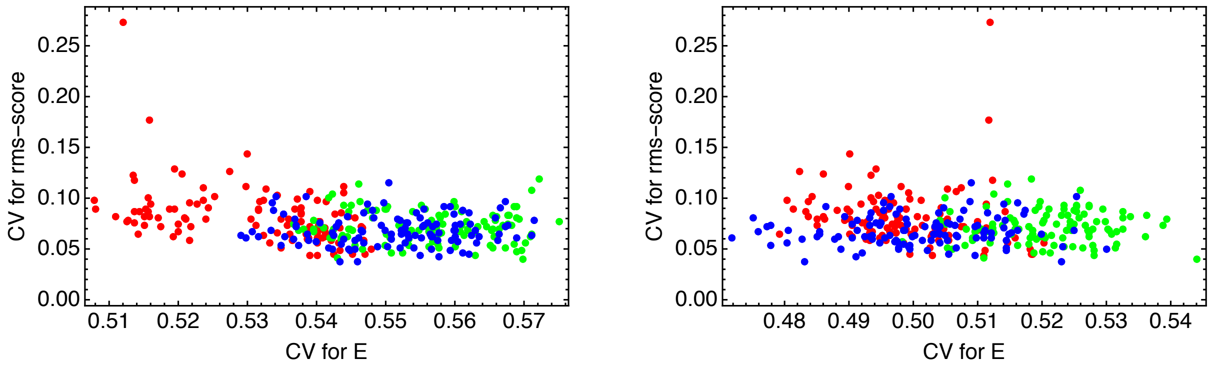

Figure S3. The same as in Figure 6C,D from the main text, but with the coefficient of variation (CV=SD/mean) instead of the mean.

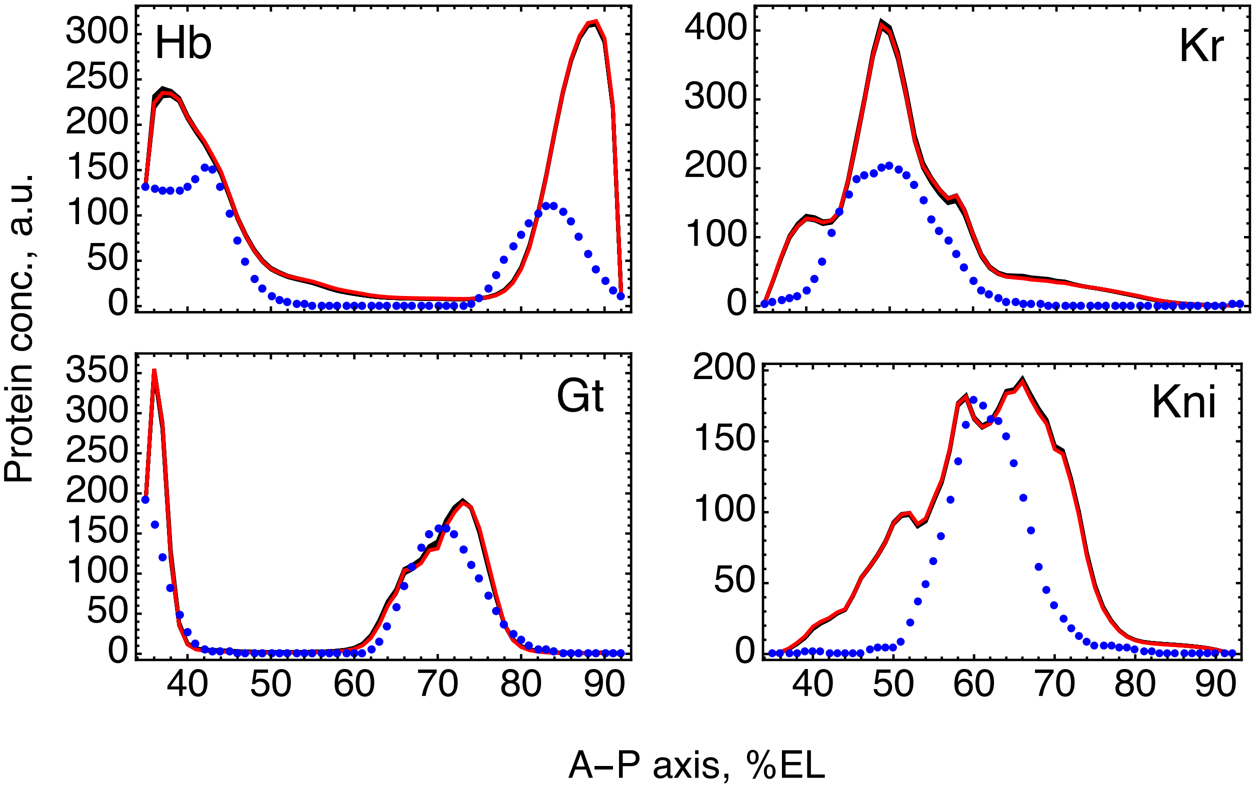

Figure S4. The expression patterns (protein concentrations) for four gap genes from the model (red and black curves) and from data (blue dots), at the end of the cleavage cycle 14A of *Drosophila* development. The red curves represent the model solution for the reference genotype (the initial genotype in the evolutionary simulation). The black curves are the model solutions for the 20 best fitted sequences from the last generation in the evolutionary simulation (they are hardly visible because of too small deviations from the red curve). The horizontal axis shows the position at the anterior-posterior (A-P) axis of the embryo in terms of percent of the embryo length.

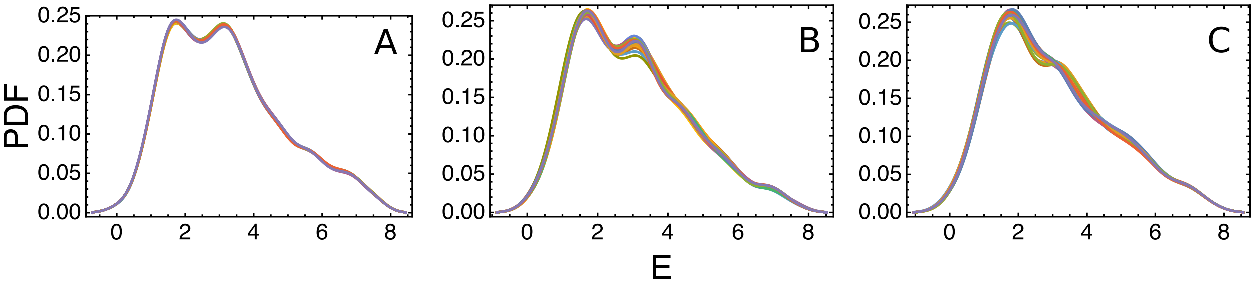

Figure S5. Distribution of the binding energy *E* for all binding sites in a sequence, for 20 best fitted sequences in generation 10 (A), 1000 (B), and 3350 (C).

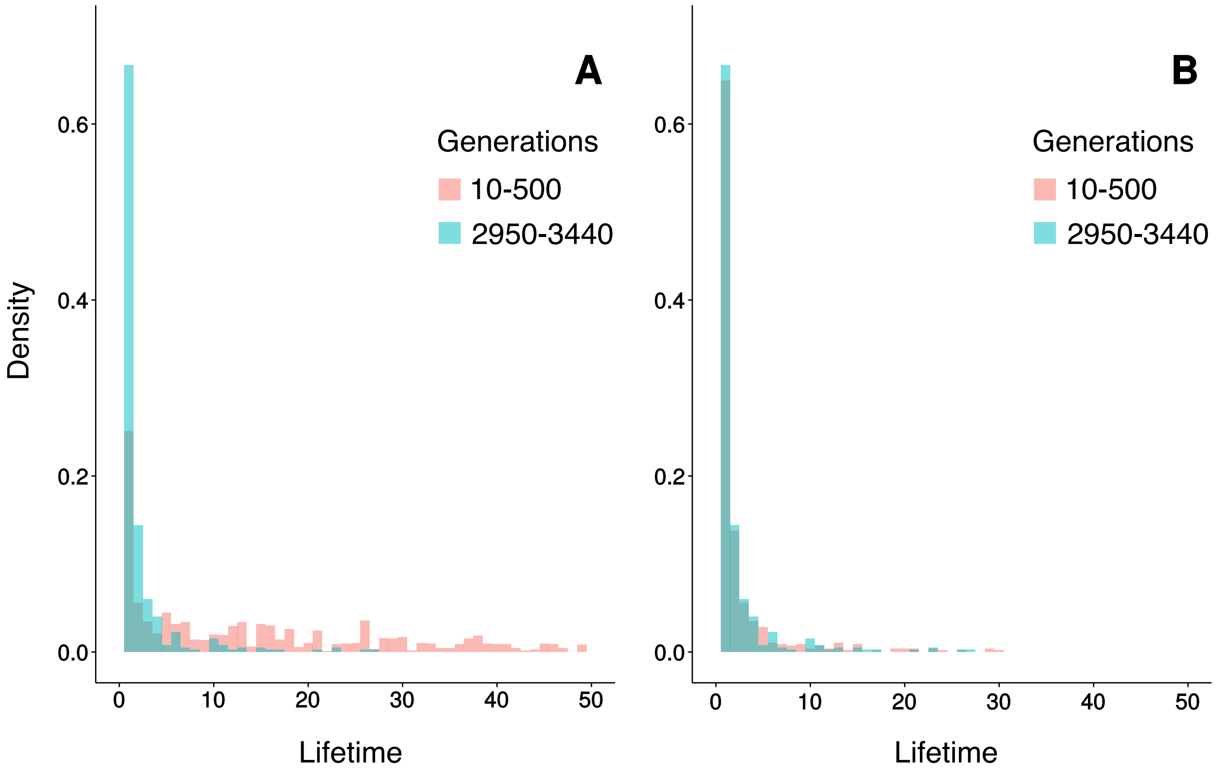

Figure S6. Distributions of lifetime of the tracked binding sites that both are born and die in the first 500 generations (red) or in the last 500 generations (green). In A, the red sample is additionally augmented with the initial binding sites that die in the first 500 generations. This makes the two distribution statistically different. In B, such initial binding sites are not taken into account, and the distributions become indistinguishable.

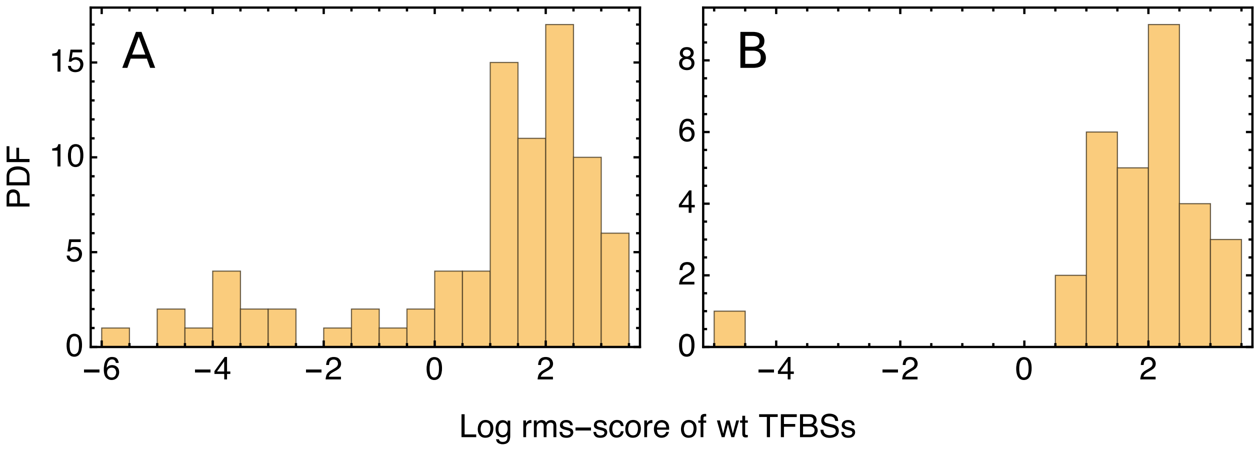

Figure S7. Distribution of the regulatory *rms*-scores of TFBSs in the model for the initial sequence, for two sets of TFBSs: all core sites (A) and the core sites that do not overlap with other core sites (B). All but one sites from the left mode of the distribution in A are involved in overlapping with core sites.

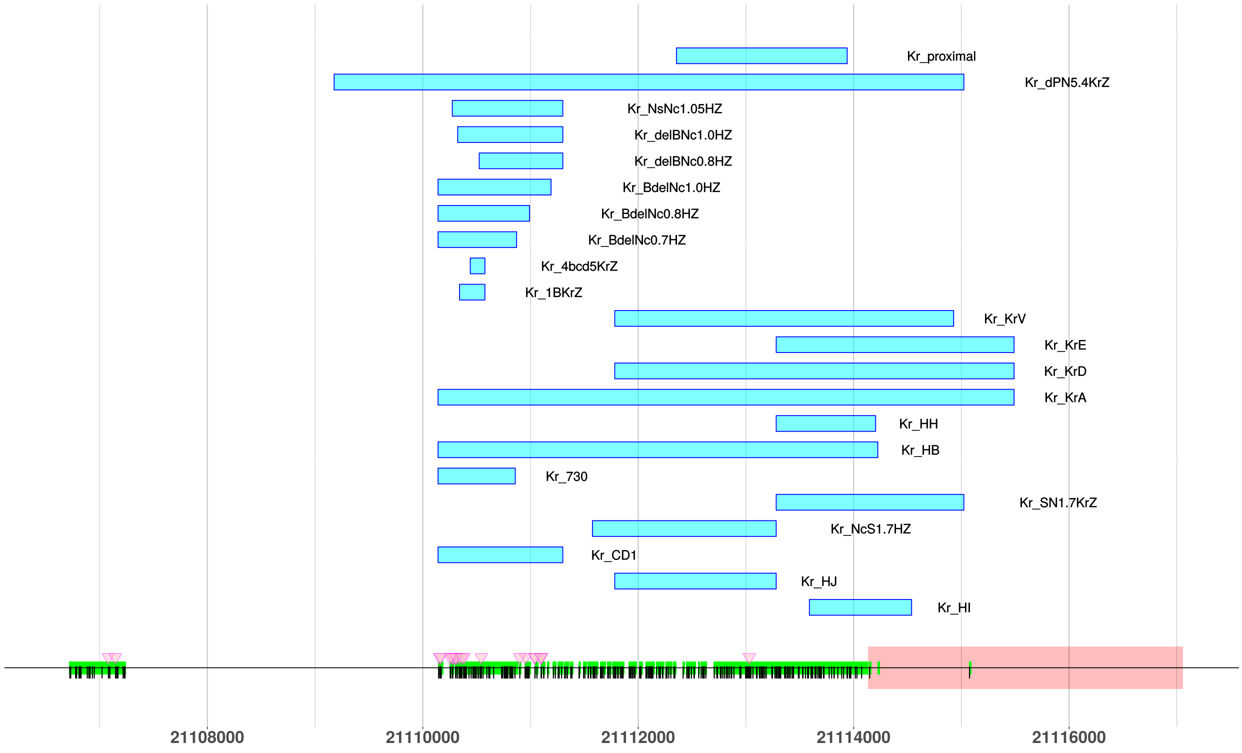

Figure S8. The organization of the regulatory region for the gene *Kruppel*. DNA sequence is shown below with the coordinates according to the dm3 assembly of the *Drosophila menalogaster* reference genome. The coding sequence is in red, and the DNAse I accessible domains are in green. The short vertical black lines on the sequence mark starting positions of TFBSs. The pink triangles show the positions of the core binding sites. The blue boxes above the sequence correspond to the placement of known enhancers, according to RedFly database.

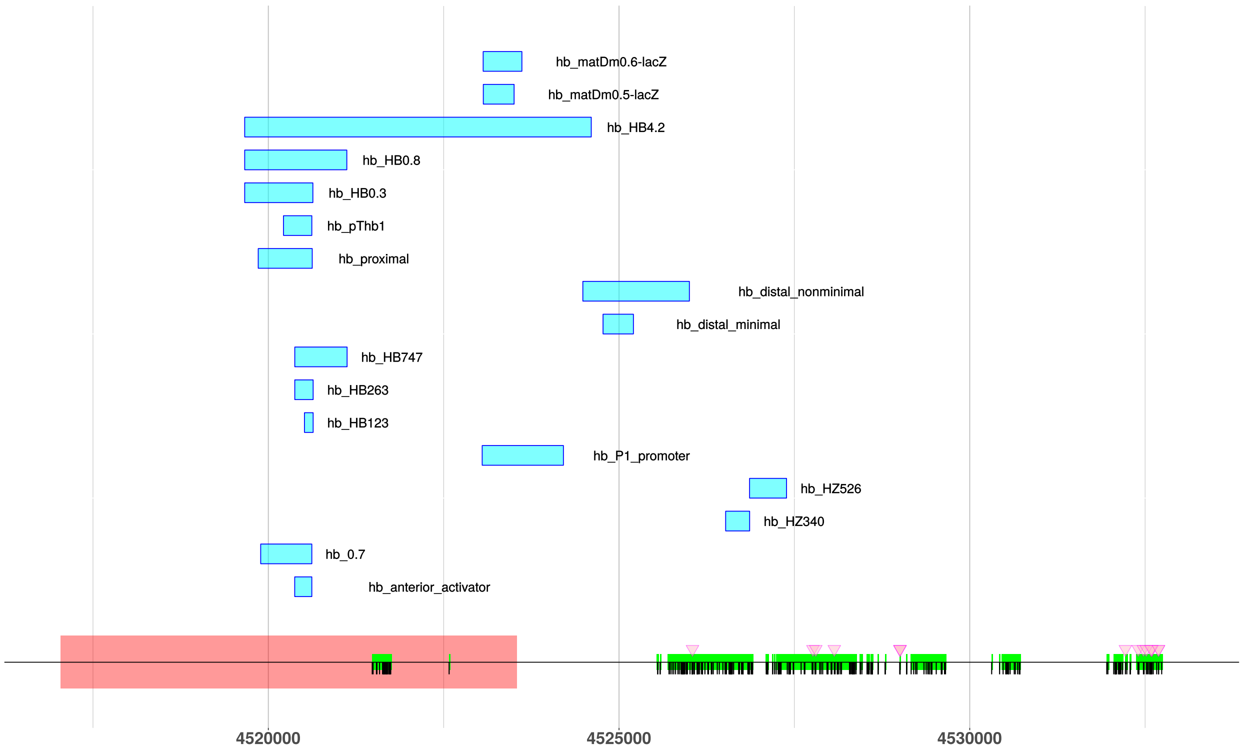

Figure S9. The same as in Figure S8, but for the gene *hunchback*.

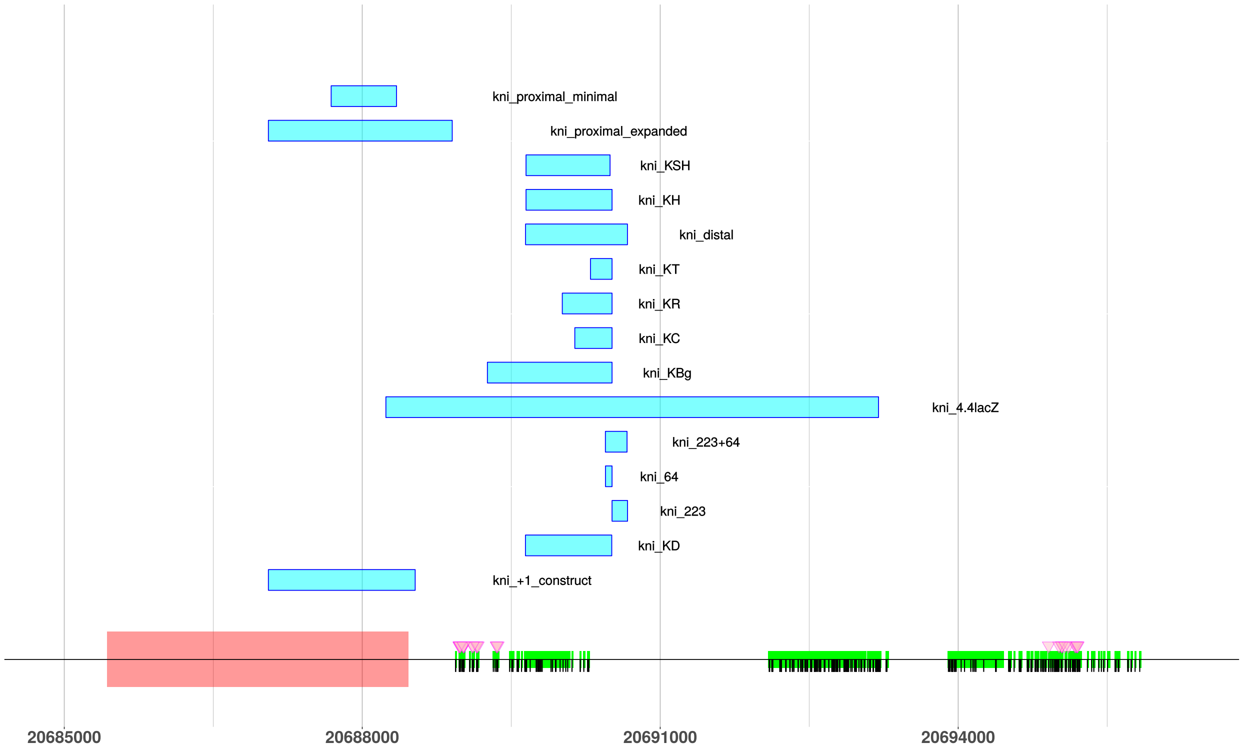

Figure S10. The same as in Figure S8, but for the gene *knirps*.

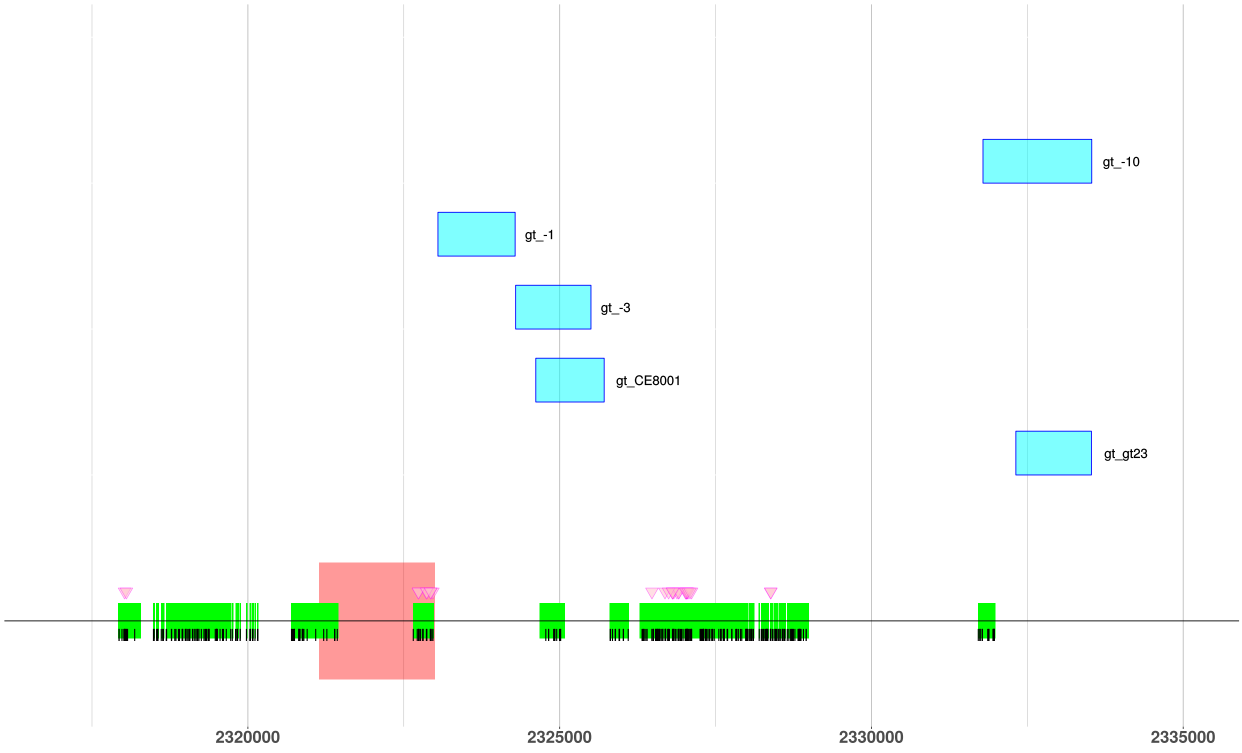

Figure S11. The same as in Figure S8, but for the gene *giant*.
